# MAFLD/NAFLD Biopsy-Free Scoring Systems for Hepatic Steatosis, NASH, and Fibrosis Diagnosis

**DOI:** 10.3389/fmed.2021.774079

**Published:** 2022-01-13

**Authors:** Nancy de los Ángeles Segura-Azuara, Carlos Daniel Varela-Chinchilla, Plinio A. Trinidad-Calderón

**Affiliations:** ^1^Tecnológico de Monterrey, School of Medicine, and Health Sciences, Monterrey, Mexico; ^2^Tecnológico de Monterrey, School of Engineering, and Sciences, Monterrey, Mexico

**Keywords:** MAFLD, NAFLD (non alcoholic fatty liver disease), scoring-algorithm, biopsy, steatosis, NASH, fibrosis, diagnosis

## Abstract

Metabolic dysfunction-associated fatty liver disease (MAFLD), formerly known as nonalcoholic fatty liver disease, is the most prevalent liver disorder worldwide. Historically, its diagnosis required biopsy, even though the procedure has a variable degree of error. Therefore, new non-invasive strategies are needed. Consequently, this article presents a thorough review of biopsy-free scoring systems proposed for the diagnosis of MAFLD. Similarly, it compares the severity of the disease, ranging from hepatic steatosis (HS) and nonalcoholic steatohepatitis (NASH) to fibrosis, by contrasting the corresponding serum markers, clinical associations, and performance metrics of these biopsy-free scoring systems. In this regard, defining MAFLD in conjunction with non-invasive tests can accurately identify patients with fatty liver at risk of fibrosis and its complications. Nonetheless, several biopsy-free scoring systems have been assessed only in certain cohorts; thus, further validation studies in different populations are required, with adjustment for variables, such as body mass index (BMI), clinical settings, concomitant diseases, and ethnic backgrounds. Hence, comprehensive studies on the effects of age, morbid obesity, and prevalence of MAFLD and advanced fibrosis in the target population are required. Nevertheless, the current clinical practice is urged to incorporate biopsy-free scoring systems that demonstrate adequate performance metrics for the accurate detection of patients with MAFLD and underlying conditions or those with contraindications of biopsy.

## Introduction

Metabolic dysfunction-associated fatty liver disease (MAFLD), formerly known as nonalcoholic fatty liver disease (NAFLD), is the most prevalent liver disorder worldwide (1, 2). Besides being considered a major public health concern (3, 4), it is expected to become the leading cause of liver failure requiring transplantation by 2030 (5).

Specifically, NAFLD is defined as an increase in hepatic lipid content not associated with chronic hepatitis due to viral infections, autoimmune diseases, or the use of steatogenic medications (6–9). Moreover, NAFLD can progress from steatosis to nonalcoholic steatohepatitis (NASH), fibrosis, and eventually, cirrhosis and hepatocellular carcinoma (10). In its early phases, the disease has a silent presentation, thus hindering the diagnosis and placing patients at risk of worse clinical outcomes (11, 12).

**Figure 1:**
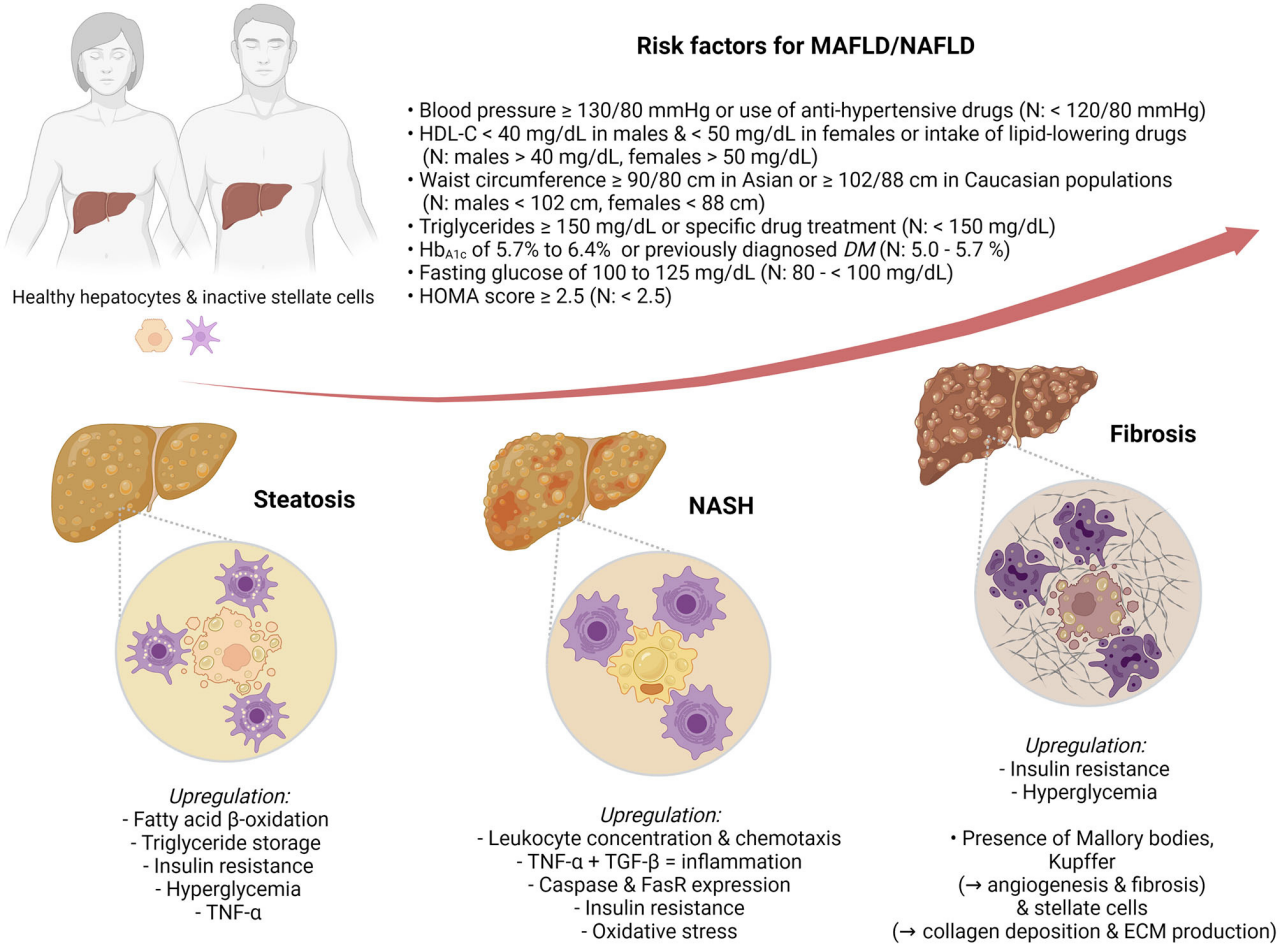
Metabolic dysfunction-associated fatty liver disease (MAFLD)/nonalcoholic fatty liver disease (NAFLD) risk factors and pathophysiological markers of hepatic steatosis (HS), nonalcoholic steatohepatitis (NASH), and fibrosis.

Nowadays, NAFLD is considered the hepatic component of metabolic syndrome (metabolic syndrome) (13), a disorder intricately related to type 2 diabetes mellitus (T2DM) (14, 15), insulin resistance, and cardiovascular diseases (16). For this reason, some authors have proposed a new, flexible term, MAFLD (17–19) (Figure 1).

Historically, MAFLD/NAFLD diagnosis required liver biopsy (20). Liver biopsy is a painful, invasive procedure that can increase mortality from 0.009 to 0.14%, has a risk of intraperitoneal hemorrhage, and only assesses approximate 1 per 50,000 of the entire liver parenchyma (21). In response, the need for new non-invasive strategies has been evidenced (22–25), especially for patients with underlying conditions (26) or biopsy contraindications (27).

Recently, grouping several non-invasive serological biomarkers has become a trend for the prediction and diagnosis of liver fibrosis (28). Moreover, studies have shown that these systems may avoid up to 38–80% of liver biopsies (29, 30). Currently, no single marker has been used for the precise detection of MAFLD/NAFLD, as isolated biomarkers do not provide sufficiently accurate information for diagnosis (31–33). However, when coupled with clinical features and with each other, accurate diagnosis, staging, and prognosis for this disease become possible (34).

Therefore, this review presents the state-of-the-art biopsy-free scoring systems (BFSS) for the diagnosis of MAFLD/NAFLD. Moreover, it further contrasts, in a stratified arrangement (Figure 1) of hepatic steatosis (HS), NASH, and fibrosis, the biomarkers, clinical associations, and discriminating performance metrics (Table 1) of such BFSS.

**Table 1:** Performance metrics and calculation formulas of biopsy-free scoring systems for metabolic dysfunction-associated fatty liver disease (MAFLD)/nonalcoholic fatty liver disease (NAFLD) staging.

**Biopsy-free scoring systems**	**Application**	**NCV**	**PCV**	**Sensitivity**	**Specificity**	**NPV**	**PPV**
**Hepatic steatosis**							
NAFLD ridge score^a^ (35, 36)	MAFLD/NAFLD	0.24	0.44	**0.91**	**0.90**	0.95	0.70
NAFLD liver fat score^b^ (36, 37)	MAFLD/NAFLD	< −0.64	> 0.64	0.86	0.71	ND	ND
Hepatic steatosis index^c^ (38, 39)	MAFLD/NAFLD	<30	> 36	**0.93**	**0.93**	0.84	0.86
Fatty liver index^d^ (40–42)	MAFLD/NAFLD	<30	> 60	0.87	0.86	ND	ND
Lipid accumulation product^e^ (36, 43, 44)	MAFLD/NAFLD	ND	ND	0.78–0.85	0.78–0.85	ND	ND
**Nonalcoholic Steatohepatitis**							
CA index^f^ (45)	NASH/Fibrosis	<10.27	> 10.27	0.81	0.83	0.92	0.63
NAFIC score^g^ (46)	NASH/Fibrosis	<1.00	> 2.00	0.63	0.64	0.69	0.36
NASH diagnostics (47)	NASH	0.20	0.34	0.77	0.87	0.73	0.89
G-NASH model^h^ (48)	NASH	ND	ND	0.73	0.32	0.59	0.54
ClinLipMet score^i^ (49)	NASH	ND	ND	0.86	0.72	0.95	0.45
**Fibrosis**							
APRI^j^ (50)	Fibrosis	<0.60	> 1.50	0.74	0.67	0.72	0.70
Fibrosis-4 index^k^ (29, 51)	Advanced fibrosis	<1.30	> 1.30	0.84	0.68	0.95	0.70
Forns index^l^ (30)	Advanced fibrosis	<4.20	> 6.90	0.29	**0.95**	0.70	0.78
BARD score^m^ (52)	Advanced fibrosis	0.1	> 3.25	0.88	0.88	0.96	0.68
NAFLD fibrosis score^n^ (53)	Fibrosis	< −1.45	> 0.67	0.82	0.77	0.93	0.93
Hepamet fibrosis score (54)	Advanced fibrosis	<0.12	> 0.47	0.74	**0.97**	0.92	0.76
Enhanced liver fibrosis test^o^ (55)	Advanced fibrosis	<7.70	> 9.80	0.74	**0.92**	0.92	0.75
Fibrometer^p^ (56)	Advanced fibrosis	0.31	0.38	0.78	**0.95**	0.92	0.87
FibroMax (57)	NASH/Fibrosis	ND	ND	0.64–0.74	0.60–0.73	0.23–0.87	0.51–0.94
**Other Biopsy-Free Scoring Systems**							
BAAT score^q^ (39, 58)	Fibrosis	0–1.00	> 2.00	0.71	0.8	0.86	0.61
Nice model (59, 60)	Advanced fibrosis	ND	0.14	0.84	0.86	0.98	0.44
OW liver test (61, 62)	NASH	<0.54	> 0.54	0.83	**0.94**	0.90	0.89
NASH score (63)	NASH	ND	2.12	0.71	0.73	0.53	0.83
GlycoNASH test (64)	NASH	ND	ND	0.67	0.64	ND	ND
Liver biopsy (65)	All	-	-	**0.93**	**0.95**	-	-

NCV, negative cutoff value; PCV, positive cutoff value; NPV, negative predictive value; PPV, positive predictive value; ND, not determined; MAFLD, metabolic dysfunction associated fatty liver disease; NAFLD, nonalcoholic fatty liver disease; NASH, nonalcoholic steatohepatitis. Bold values denote figures of sensitivity and specify above 0.90.

Calculation formulas:

a *NRS: −0.614 + 0.007 × ALT −0.214 × HDLC + 0.053 × triglycerides + 0.144 × HbA1c + 0.032 × WBC + 0.132 × hypertension*.

b *NLFS: 1.18 × MS + T2DM (2 if yes; 0 if no) + 0.15 × fasting insulin (mU/L) + 0.04 × AST(U/L) 0.94 × (AST/ALT) 2.89*.

c *HSI:8 × (ALT/AST ratio) + BMI (+2, if female; +2, if T2DM)*.

d *$FLI:\;e^{0.953}\times {Log_{e}}^{\left(TG\right)}+0.139\times BMI+0.718\times {Log_{e}}^{\left(GGT\right)}+0.053\times \;WC-15.745/\left[1+\;e^{0.953}\times \;{Log_{e}}^{\left(TG\right)}\;+0.139\times BMI+0.718\times {Log_{e}}^{\left(GGT\right)}+0.053\times \;WC-15.745\right]\times 100$*.

e *LAP: (WBC 65) × triglycerides if male; (WBC 58) × triglycerides if female*.

f *CA: (0.994 × type IV collagen 7S + 0.0255 × AST)*.

g *NAFIC: (ferritin ≥ 200 ng/mL [female] or ≥ 300 ng/mL [male]:1 point) + (fasting insulin ≥ 10 lU/mL:1 point) + (type IV collagen 7 s ≥ 5.0 ng/mL:2 points)*.

h *G − NASH: 0.02 × GP73 (ng/ml) + 0.123 × AST (U/L) + 0.1576 × zinc (μmol/L) + 0.0227 × total thyrosine (nmol/L) − 0.4525 × SDPV (fL) + 2.0789 × (BMI ≥ 30 kg/m2, yes = 1, no = 0)*.

i *ClinLipMet: − 0.305 + 0.562 × PNPLA3 genotype (CC − 1/GC − 2/GC − 3) 0.0092 × fasting insulin (mU/L) + 0.0023 × AST (IU/L) + 0.0019 × (fasting insulin × AST)*.

j *APRI: {AST (IU/1)/[upper normal value of 41 (IU/l)]}/platelets (× 10^9^/l) × 100*.

k *$FIB-4:\;age\;\times \;AST\;\left(IU/l\right)/platelets\;\left(\times \;109/l\right)\;\times \;\sqrt{}\;ALT\;\left(IU/l\right)$*.

l *Forns: 7.811 − 3.131 × ln(platelets) + 0.781 × ln(GGT) + 3.467 × ln(age) − cholesterol*.

m *BARD: (BMI > 28 = 1 point) + (AAR > 0.8 = 2 points) + (DM = 1 point)*.

n *NFS: 1.675 + [0.037 × age] + [ 0.094 × BMI (kg/m^2^)] + [1.13 × abnormal FGL or T2DM (yes = 1, no = 0)] + [0.99 × AAR] [0.013 × platelets (× 10^9^/l)] [0.66 × albumin (g/dl)]*.

o *ELF: 2.494 + 0.846 ln(HA) + 0.735 ln(PIIINP) + 0.391 ln(TIMP1)*.

p *Fibrometer: 0.4184 glucose (mmol/L) + 0.0701 AST (IU/L) + 0.0008 ferritin (μg/L) − 0.0102 platelet (G/L) − 0.0260 ALT (UI/L) + 0.0459 body weight (kg) + 0.0842 age + 11.6226*.

q *BAAT: (BMI ≥ 28 = 1 point) + (age ≥ 50 years = 1 point) + (ALT ≥ 2N (1 point)) + (triglycerides ≥ 1.7 mmol/L (1 point))*.

## Hepatic Steatosis Scoring Systems

Defined as a lipid concentration >5% in the hepatic parenchyma (66) without portal or lobular inflammation (67), HS is the mildest form of MAFLD/NAFLD (68). Currently, 4% of patients with HS are expected to develop fibrosis in their lifetimes (69). Thus, the BFFS proposed to aid in the prompt diagnosis are discussed in this section.

### NAFLD Ridge Score

This BFSS considers alanine aminotransferase (ALT), hemoglobin A_1C_, high-density lipoprotein C, hypertension, leukocyte count, and triglycerides (35). The enzyme ALT level increases in serum as hepatocytes are damaged (36). Similarly, high levels of triglycerides, low levels of high-density lipoprotein C, hypertension, and increased hemoglobin A_1C_ level correlate with HS (70, 71). Moreover, increased intrahepatic leukocyte concentration is associated with the progression to NAFLD risk factors and stage-specific markers of NASH (72, 73).

Notably, this score has an area under the receiver-operating curve (AUROC) of 0.87 (74). Nevertheless, it is unreliable for distinguishing steatosis grades (36) and ends up classifying as indeterminate up to 30% of patients (35).

### NAFLD Liver Fat Score

Developed in a Finnish population (37), this BFSS weighs aspartate aminotransferase (AST), AST/ALT ratio, fasting insulin, metabolic syndrome, and T2DM (75). Insulin levels correlate with HS grades, as insulin resistance is an important risk factor for the development of MAFLD/NAFLD (70). Moreover, AST levels increase as AST is released from injured hepatocytes, indicating liver dysfunction (36).

This BFSS can predict MAFLD/NAFLD and estimate the liver fat contents >5.56%, with an AUROC of 0.88 (36, 37). Moreover, it has shown a positive correlation with the incidence and mortality of cardiovascular disease, which are outcomes intricately related to metabolic syndrome and T2DM (76). Nonetheless, this score has a poor capacity for quantifying steatosis, as its AUROC for predicting >33% of steatosis significantly decreases at 0.72 (77).

### HS Index

This index assesses MAFLD/NAFLD (78) on the basis of body mass index (BMI), AST/ALT ratio, and the presence of T2DM (38). AST/ALT ratio is used to assess the HS grade more accurately than any of its components individually (79). Similarly, both enzymes positively and almost linearly correlated with increased incidence of MAFLD/NAFLD and premature mortality risk (80). In addition, studies have reported that this test has an AUROC of 0.75 (78, 81). Moreover, this BFSS has a high correlation with HS grades diagnosed using ultrasonography, but this score has not yet been validated for NASH (38).

### Fatty Liver Index

Created as an algorithm to detect fatty liver (40), this index is based on BMI, gamma glutamyl transferase (GGT), triglycerides, and waist circumference (82). Waist circumference correlates with visceral adiposity, an important predictor of metabolic syndrome (83). Similarly, the accumulation of triglycerides in hepatocytes produces hepatocyte ballooning and inflammation, both changes associated with MAFLD/NAFLD (84). High levels of GGT, in particular, are associated with increased incidence rates of hypertension and insulin resistance (85).

The BFSS has an AUROC of 0.82 for MAFLD/NAFLD detection (86). However, it was validated only in certain populations, such as Koreans (82), Chinese (87), and Northern Italians (40).

### Lipid Accumulation Product

The BFSS is used to evaluate waist circumference and triglyceride levels (43). Distinctively, it has been adjusted for age, sex, and ethnicity (88). This score is only validated in a cohort in Northern Italy (89). Although it was originally developed as a reference for cardiometabolic risk, it was later validated as an HS index (36, 44).

Furthermore, it has an AUROC, 0.77 for NAFLD diagnosis and was more accurate in patients with hypertriglyceridemia (AUROC, 0.73) compared with patients with T2DM (AUROC, 0.67) (86). However, even if the BFSS can detect MAFLD/NAFLD clinically, its main limitation is in distinguishing patients with mild disease from those with more severe MAFLD/NAFLD (90).

## NASH Scoring Systems

Nonalcoholic steatohepatitis consists of fatty liver in conjunction with inflammation and hepatocellular injury, with or without fibrosis (91). More than 20% of patients with NASH are expected to develop cirrhosis in their lifetimes (69). Consequently, this section delves into the BFSS proposed for its detection (92, 93).

### CA Index

This index owes its name to its two parameters, type IV collagen 7S and AST. Specifically, type IV collagen 7S is an indirect marker of fibrogenesis (94) and AST reiterates its role in liver dysfunction (36). Currently, the BFSS is used to predict NASH and fibrosis, with AUROC of 0.85 and 0.91, respectively (95). Moreover, it identifies MAFLD/NAFLD without fibrosis and NASH-related fibrosis (94, 96). Unfortunately, the CA index was only validated in the Japanese population, similarly to the NAFIC score (97).

### NAFIC Score

This score is based on ferritin, fasting insulin, and type IV collagen 7S levels (24, 98). Comparatively, the BFSS is used for evaluating ferritin levels, which increases in patients with NASH (99). Similarly, fasting insulin is considered as a correlation marker for HS (70), and type IV collagen 7S is used, as in the CA index (100).

The BFSS has an AUROC of 0.85 and 0.83 for NASH and fibrosis, respectively (46), both higher than the BARD [0.76 (101)] and NAFLD fibrosis score [0.77 (102)]. Nevertheless, such accuracy has been only validated in Japanese patients (46, 103).

### NASH Diagnostics

This biomarker panel is used to diagnose obesity-related NASH based on adiponectin, cleaved cytokeratin 18 (CK-18) M30, and resistin levels (47). Adiponectin is inversely correlated with the risk of metabolic syndrome (104). Similarly, CK-18 M30 is proposed as a differentiator between NASH and MAFLD/NAFLD without inflammation (24, 105). Finally, resistin has been associated with obesity, insulin resistance, and T2DM (106, 107).

The BFFS has a reported AUROC value of 0.90 (47). However, it requires further validation in cohorts other than morbidly obese candidates for bariatric surgery (108). Similarly, a major limitation of its specificity is possibly due to all three of its parameters being increased in various liver diseases (106, 109), thus making them nonspecific markers of NASH (110, 111).

### G-NASH Model

This novel BFSS is based on AST, BMI, CK-18 M30, Golgi protein 73, platelets, thyroxine, and zinc (48). Specifically, CK-18 M30 fragments increase in patients with MAFLD/NAFLD and T2DM (112), and correlate positively with high ALT, glucose, and hemoglobin A_1C_ levels, systolic blood pressure, and triglyceride levels (113). Similarly, Golgi protein 73, which is only expressed in fibrotic and diseased liver tissue, is considered a promising marker of liver inflammation (114).

When grouped (48), these biomarkers identified NASH in patients with MAFLD/NAFLD who had normal ALT levels and those requiring liver biopsy, with an AUROC of 0.85 (48). Nonetheless, the BFSS lacks external validation in other populations and studies to determine its validity for screening patients at risk of developing NASH (48).

### ClinLipMet Score

Although it was only tested in Finnish and Belgian Caucasian and morbidly obese populations (49), the BFSS identified patients with NASH, with an AUROC of 0.866 (115). It considers AST and fasting insulin levels; PNPLA3 genotype rs738409, a polymorphism closely associated with increased hepatic fat content (116); and amino acid and phospholipid levels (49).

The levels of Glu, Gly, and Ile amino acids increase during progression to NASH (117). By contrast, phospholipids lysophosphatidylcholine 16:0 and phosphoethanolamine 40:6 are used to determine alterations in cell membrane metabolism in patients with advanced MAFLD/NAFLD and a higher liver fibrosis stage (118, 119). Specifically, these two molecules significantly differentiate NASH from HS but fail to do so in patients with HS and controls (49).

## Hepatic Fibrosis Scoring Systems

Chronic injury to liver myofibroblasts is known to induce fibrosis (120). In this regard, the risk of advanced fibrosis in patients with MAFLD/NAFLD is noteworthy (7.5%), along with other liver-related complications and eventually death (52, 121, 122). Correspondingly, the BFSS proposed for the diagnosis of liver fibrosis is scrutinized herein.

### AST-to-Platelet Ratio Index

The BFSS is based on AST and platelets, both of which increase in the hepatic sinusoids of patients with MAFLD/NAFLD (123, 124). In addition, it detects advanced fibrosis in patients with chronic hepatitis C virus infection (125) and is later validated for the detection of MAFLD/NAFLD (126).

The AST-to-platelet ratio index (APRI) is considered a good predictor of advanced fibrosis in patients with MAFLD/NAFLD, having an AUROC of 0.71 and 0.79 in non-bariatric and bariatric patients, respectively (127). Notwithstanding, some authors have argued against its widespread use, mainly because of its low accuracy in staging fibrosis (128, 129).

### Fibrosis-4 Index

This index had been validated for the assessment and detection of liver fibrosis based on age, ALT level, AST level, and platelet count (130, 131). Platelet count correlates with hepatocyte ballooning, fibrosis, and liver steatosis (123, 124).

Overall, the BFSS has an AUROC ranging from 0.80 to 0.86 (128). Specifically for non-bariatric and bariatric patients, it has an AUROC of 0.83 and 0.81, respectively, which are higher than those obtained for APRI (0.71 and 0.79, respectively) (127). Nonetheless, certain studies have argued that the inclusion of age might lead to a falsely worse score in the elderly population and thus increase the false-positive rate (132).

### Forns Index

This index is based on platelet count, cholesterol level, GGT levels, and age (133, 134). The importance of this index relies on GGT, which has been associated with insulin resistance (85), and on cholesterol, which correlates negatively with the liver fibrosis stage, thus aiding in NASH diagnosis (30). In this regard, the BFSS is used as a predictor of advanced fibrosis in patients with chronic hepatitis C virus infections, with an AUROC of 0.79 (30, 105, 134, 135). Notwithstanding, information regarding its accuracy in MAFLD/NAFLD is limited (30).

### BARD Score

The BARD score is based on BMI, AST/ALT ratio, and T2DM, all of which are markers of metabolic syndrome (61). Along with the NAFLD fibrosis and FIB-4 scores, the BFSS is validated for the detection of advanced fibrosis or cirrhosis, with an AUROC of 0.76 (101, 130). Even so, its low positive predictive value of 0.42 has limited its use in clinical practice (122). Nonetheless, its high reported negative predictive value of 0.96 makes the BARD score a reliable tool for ruling out advanced fibrosis (52).

### NAFLD Fibrosis Score

The BFSS is currently used to predict advanced fibrosis (53), with an AUROC of 0.77 (102), and includes age, hyperglycemia, BMI, platelet count, albumin level, and AST/ALT ratio as parameters (136). Specifically, the albumin binding function and quantity are decreased in patients with long-standing MAFLD/NAFLD (137).

A high score (>0.68) significantly correlated with a 4-fold higher risk of death in patients with MAFLD/NAFLD (5). Nevertheless, this score has a limited value in predicting changes in fibrosis, even when it accurately predicts morbidity and mortality in all stages of fibrosis (138).

### Hepamet Fibrosis Score

This novel BFSS is based on age; albumin, AST, and glucose levels; homeostatic metabolic assessment, which positively correlated with a higher stage of liver fibrosis and stiffness (139); insulin level; platelet count; sex; and T2DM (54, 140). It has a high accuracy for advanced fibrosis exclusion (30), with a reported AUROC value of 0.94 for advanced fibrosis prediction (30). Even so, this score had confounding results in patients with T2DM (141), a finding that created uncertainty because more than 70% of such patients concomitantly have MAFLD/NAFLD (142).

### Enhanced Liver Fibrosis Test

This test is based on the levels of hyaluronic acid, type III procollagen peptide, and the tissue inhibitor of metalloproteinase 1 (143). Their concentrations and activities make this test useful for grading liver fibrosis (144, 145). In addition, studies have shown that the BFSS is an accurate tool for detecting advanced fibrosis in patients with MAFLD/NAFLD (146), mainly owing to its AUROC of 0.85 for stage F2 and 0.90 for stage F3 with NASH (147). Recently, a meta-analysis revealed that this fibrosis test has a high sensitivity for advanced fibrosis, but a limited specificity in low-prevalence areas (148).

### FibroMeter

On the basis of markers, such as age, ALT level, AST level, body weight, ferritin level, glucose level, and platelet counts (149). FibroMeter identifies fibrotic areas and fibrosis stage (150), with higher reproducibility when compared with other diagnostic tools (149). Quantitatively, FibroMeter has AUROC values of 0.94, 0.93, and 0.9 for significant fibrosis, advanced fibrosis, and cirrhosis, respectively (58, 149). Furthermore, its results for fibrotic areas have an AUROC of 0.94, which is more accurate in comparison with that of the NAFLD fibrosis score (0.88) and APRI (0.87) (7, 7, 149, 151, 152). Nonetheless, some authors argued that ethnicity-specific cutoff values would increase its validity (153).

### FibroMax

FibroMax is a BFSS that combines five components into one algorithm (154). Among the components, ActiTest showed a significant accuracy in NASH diagnosis and MAFLD/NAFLD differentiation (155). It is considered as an accurate score for liver fibrosis (154, 156), with an AUROC of 0.68 for grade 2 and 3 steatosis, 0.59 for NASH, and 0.79 for fibrosis (157).

Furthermore, studies reported that FibroTest, another component of FibroMax, had higher accuracy in discriminating severe fibrosis stages and detecting cirrhosis than low to intermediate stages (158). FibroTest is not accurate for differentiating between the zonal distribution of fibrosis in MAFLD/NAFLD; thus, its effectiveness has been controversial (156).

Nonetheless, both components are affected by acute hemolysis, inflammation, and extrahepatic cholestasis (51). Similarly, in response to its low AUROC, they are considered unreliable alternatives for liver biopsy in MAFLD/NAFLD (157).

## Discussion

Numerous authors have proposed biopsy-free scoring systems as screening tools for fatty liver and risk-stratifying systems based on fibrosis (51, 144, 159) for the MAFLD/NAFLD spectrum (95). Nonetheless, they still emphasize the importance of liver biopsy as the diagnostic standard but urge for a clear identification of biopsy indications (conflicting clinical or serological data), an issue that can be addressed with noninvasive diagnostic tools, such as BFSS (160–162). Some BFSSs addressed in this review (G-NASH, ClinLipMet, and enhanced liver fibrosis test) measure components that are not readily available, seldom ordered, or expensive, such as the PNPLA3 genotype, CK-18 M30 fragments, Golgi protein 73, or the tissue inhibitor of metalloproteinase 1. Comparatively, other scores, such as the lipid accumulation product, fatty liver index, HS index, APRI, fibrosis-4 index, Forns index, and NAFLD fibrosis score rely on routinely ordered components, thus facilitating their use. Furthermore, as patients develop more metabolic abnormalities, they tend to yield higher scores (163, 164), making these BFSSs more reliable as the condition of the patient worsens. However, some scores have been validated only in limited populations, such as the CA index (97), fatty liver index (40, 82, 87), and NAFIC score (46, 103), whereas others are inaccurate for MAFLD/NAFLD staging [FibroMax (157)] or when associated with other comorbidities [Hepamet fibrosis score (142)]. These limitations must be addressed through validation in other populations (97), with attention to variables, such as BMI, comorbidities, and ethnicity (49, 125, 143, 165–167). Comparatively, other BFSSs have been shown to have high sensitivity, such as the NAFLD ridge score (35, 36) or HS index (38, 39), and specificity, such as the Hepamet fibrosis score (54), Forns index (30), and enhanced liver fibrosis test (55), making them accurate tests for screening and confirmation of disease, respectively. Certain BFSSs underperformed in validation studies, such as the BAAT score (168), Nice model (59, 60), OW liver test (61, 62), NASH score (63), CHeK model (165), or GlycoNASH test (64), making them unsuitable alternatives for MAFLD/NAFLD diagnosis; thus, they were consequently excluded from the scrutiny of this review. Comprehensive studies on the effects of age, BMI, obesity, and the prevalence rates in different populations (101, 140, 148, 169) are required to determine the role of current and future BFSSs in MAFLD/NAFLD diagnosis. Other non-invasive alternatives have been proposed recently, such as cell-free DNA, which has been found in extracellular vesicles in the serum of patients with fatty liver, and have yielded promising results (170). Moreover, novel considerations, such as the addition of enhanced liver fibrosis test to clinical practice guidelines (171, 172) will eventually play a larger role in the diagnosis and follow-up of patients. As more information is gathered, novel considerations will be implemented, aiding in a more precise understanding and accurate detection of MAFLD/NAFLD in the global population (173).

## Concluding Remarks

Clinicians are urged to include BFSS for the diagnosis of early stages of MAFLD/NAFLD, particularly in patients with a high risk of liver fibrosis, even if these are still outperformed by biopsy in terms of accuracy. Increasing the awareness of the available BFSSs for staging is paramount to improving patient safety. The ever-growing MAFLD/NAFLD pandemic urges clinicians to seek alternatives for screening, early diagnosis, and follow-up, especially for those with contraindications for liver biopsy.

## Author Contributions

NS-A, CV-C, and PT-C contributed to the conceptualization of this manuscript and its graphic elements, wrote and revised the original draft, and contributed to the discussion, abstract, and final version of the manuscript. CV-C further contributed to the revision, completion, and content improvement of the manuscript. PT-C further oversaw the general progress of the study, initial revision of the manuscript, structuring of the draft, and final revision of the manuscript, figures, and tables. All authors revised and agreed to the final version of the manuscript.

## Funding

This work was supported by the Medical Publications and Conferences Support Fund 0020201D10 from Tecnológico de Monterrey.

## Conflict of Interest

The authors declare that the research was conducted in the absence of any commercial or financial relationships that could be construed as a potential conflict of interest.

## Publisher's Note

All claims expressed in this article are solely those of the authors and do not necessarily represent those of their affiliated organizations, or those of the publisher, the editors and the reviewers. Any product that may be evaluated in this article, or claim that may be made by its manufacturer, is not guaranteed or endorsed by the publisher.
